# The association between autistic traits and quality of social interactions in the daily life of adolescents and young adults

**DOI:** 10.1192/j.eurpsy.2023.246

**Published:** 2023-07-19

**Authors:** L. Fusar-Poli, T. Investigators, J. van Os, B. P. Rutten, S. Guloksuz

**Affiliations:** 1 Department of Brain and Behavioral Sciences, University of Pavia, Pavia, Italy; 2 Department of Psychiatry and Neuropsychology, School for Mental Health and Neuroscience, Maastricht University, Maastricht; 3 UMC Utrecht Brain Centre, University Medical Centre Utrecht, Utrecht University, Utrecht, Netherlands; 4 Department of Psychosis Studies, Institute of Psychiatry, Psychology & Neuroscience, King’s College London, London, United Kingdom; 5 Department of Psychiatry, Yale School of Medicine, New Haven, United States

## Abstract

**Introduction:**

Autism spectrum disorder (ASD) is a heterogenous groups of neurodevelopmental conditions characterized by difficulties in social communication and the presence of restricted interests and repetitive behaviors. Autistic traits are distributed along a continuum in the general population and are negatively associated with social functioning also in non-autistic subjects. Several studies have evaluated the association between autistic traits and the quantity of social interaction; however, evidence on the relationship between autistic traits and quality of social interaction is still scarce.

**Objectives:**

To evaluate the association between autistic traits and the quality of social interactions in daily life in youths from the general population using the experience samplic method (ESM).

**Methods:**

During a six-day experience sampling period, 349 twins and 248 of their siblings aged between 15 and 34 reported the quality of their everyday social interactions. Autistic traits were assesed using the Autism Spectrum Quotient (AQ). The association between autistic traits and quality of social interaction was tested in separate multilevel linear and logistic regression models.

**Results:**

When participants were alone, higher autistic traits were associated with a sense of being less safe (B=-0.02, p=0.02). When participants were in company, higher autistic traits were associated with a higher preference for being alone (B=0.02, p<0.001) and higher sense of being judged (B=0.03, p=0.001). Moreover, while in company, higher autistic traits were associated with a decreased pleasure of being in company (B=-0.03, p<0.001), a lower sense of being safe in company (B=-0.03, p<0.001), and a lower sense of belonging to a group (B=-0.02, p<0.001).

**Conclusions:**

The preliminary results of the present study showed that autistic traits may influence the quality of social interactions in daily life. Future studies may clarify the mechanisms underlying this association. Assessing autistic traits in youth may help improve the outcome of psychosocial interventions of youths presenting difficulties in social interactions.

**Disclosure of Interest:**

None Declared

